# miR-126 Regulation of Angiogenesis in Age-Related Macular Degeneration in CNV Mouse Model

**DOI:** 10.3390/ijms17060895

**Published:** 2016-06-07

**Authors:** Lei Wang, Amy Yi Wei Lee, Jonathan P. Wigg, Hitesh Peshavariya, Ping Liu, Hong Zhang

**Affiliations:** 1 Eye Hospital, First Affiliated Hospital, Harbin Medical University, Harbin 150001, China; wangleiPHD@126.com; 2 Department of Pharmacology and Therapeutics, Drug Delivery Unit, Centre for Eye Research Australia, University of Melbourne, East Melbourne VIC 3000, Australia; amylee1192@gmail.com; 3 Centre for Eye Research Australia, Royal Victorian Eye and Ear Hospital, University of Melbourne, East Melbourne VIC 3000, Australia; jonathanwigg@outlook.com (J.P.W.); hiteshp@163.com (H.P.)

**Keywords:** miR-126, age-related macular degeneration, choroidal neovascularization, human microvascular endothelial cell (HMEC)

## Abstract

miR-126 has recently been implicated in modulating angiogenic factors in vascular development. Understandings its biological significance might enable development of therapeutic interventions for diseases like age-related macular degeneration (AMD). We aimed to determine the role of miR-126 in AMD using a laser-induced choroidal neovascularization (CNV) mouse model. CNV was induced by laser photocoagulation in C57BL/6 mice. The CNV mice were transfected with scrambled miR or miR-126 mimic. The expression of miR-126, vascular endothelial growth factor-A (VEGF-A), Kinase insert domain receptor (KDR) and Sprouty-related EVH1 domain-containing protein 1 (SPRED-1) in ocular tissues were analyzed by qPCR and Western blot. The overexpression effects of miR-126 were also proven on human microvascular endothelial cells (HMECs). miR-126 showed a significant decrease in CNV mice (*p* < 0.05). Both mRNA and protein levels of VEGF-A, KDR and SPRED-1 were upregulated with CNV; these changes were ameliorated by restoration of miR-126 (*p* < 0.05). CNV was reduced after miR-126 transfection. Transfection of miR-126 reduced the HMECs 2D-capillary-like tube formation (*p* < 0.01) and migration (*p* < 0.01). miR-126 has been shown to be a negative modulator of angiogenesis in the eye. All together these results high lights the therapeutic potential of miR-126 suggests that it may contribute as a putative therapeutic target for AMD in humans.

## 1. Introduction

Age-related macular degeneration (AMD) is the leading cause of blindness in developed countries, affecting more than 50 million people over the age of 60 worldwide [1,2]. AMD can be categorized into two types: “dry type” or atrophic and “wet type” or neovascular. The “wet” form of AMD is associated with severe vision loss involving the progressive growth of abnormal, permeable blood vessels from the choroid into the subretinal space, known as Characterized by choroidal neovascularization (CNV) [3,4]. Excessive growth and secondary hemorrhage of these immature blood vessels lead to deterioration of the macula resident photoreceptors and if left untreated, permanent loss of vision can occur [5,6].

Organisms require an appropriate balance of stability and reversibility in gene transcription and translation to maintain cell identity or to enable responses to stimuli. This process is tightly regulated by proteinous and nonproteinous complexes such that, even slight dysregulation of this network results in the occurrence of several human diseases [7]. Ocular neovascularization is a very complicated pathophysiologic process, which is tightly regulated by the balance between the angiogenic stimulators such as vascular endothelial growth factor (*VEGF*), insulin-like growth factor (*IGF*) and angiogenic inhibitors such as pigment epithelium derived factor (*PEDF*) [8]. CNV formation associated with “wet” AMD is the outcome of dysregulation of this finely tuned angiogenesis process [9]. The stimuli that lead to the pathogenesis of AMD are believed to be multifactorial, involving an intricate correlation between genetic, environmental, metabolic and inflammatory factors [6]. Anti-*VEGF* treatment has been used widely in treating neovascular AMD. However, the administration of anti-*VEGF* therapy has led to some severe side effects such as stroke [10]. Moreover, VEGF inhibitors have shown to be ineffective in a subset of AMD patients [11]. The opportunity exists for the development of new therapies.

miRNAs are short non-coding RNA molecules that consist of about 22 nucleotides. They play a vital role in regulatory mechanisms of complex physiological and pathological processes by post-transcriptionally modulating gene expression, generally by translational repression or degradation of mRNA [12,13]. Since their discovery, miRNAs have been implicated in the pathophysiological processes of various diseases such as cancer and cardiovascular diseases [14,15]. Recent studies have revealed that a number of miRNAs were involved in the process of angiogenesis [16,17,18], and several miRNAs have also been shown to be distinctly involved in new vascular development in the retina [19,20,21]. *miR-31*, *miR-150* and *miR-184* have shown to be downregulated in oxygen-induced retinopathy mice models [20]; *miR-23~24~27* cluster was upregulated in laser induced CNV mice models [21]. These studies suggest a potential involvement of miRNAs in the development and progression of wet AMD [6,20], and further examination into the role of miRNAs may provide insight into potential treatments for this disease.

Among these miRNAs, *miR-126* is a likely candidate for involvement in pathogenic neovascularization. *miR-126* is an EC-specific miRNA encoded in the intron of *EGF-like domain 7* and has been shown to be involved in tumor neovascularization [22,23]. *VEGF* plays a pivotal role in modulating endothelial cell function, such as blood vessel formation during embryonic development. Some studies indicated that *miR-126* regulates tumor angiogenesis by targeting VEGF-A [24]. In endothelial cells, signaling through *VEGF*-receptor 2 (*VEGFR2*) promotes cell survival [25]. Kinase insert domain receptor (*KDR*), which encodes *VEGFR2*, also plays an important role to promote angiogenesis. Recently, Sprouty/Spred family proteins were identified as negative regulators for growth factor- and cytokine-induced *ERK* activation in angiogenesis [26,27,28,29]. In one study, miR-126 was found to regulate angiogenic signaling and vascular integrity by targeting Sprouty-related *EVH1* doain-containing protein 1 (*SPRED-1*) and phosphoinositol-3 kinase regulatory subunit 2 (*PIK3R2*) [19]. In ocular neovascular diseases, endothelial cells respond to various proteins and growth factors that regulate angiogenesis [9]. However, there have been contradicting reports on the roles of *miR-126*. Fish *et al.* and Wang *et al.* proved that *miR-126* enhanced angiogenesis [19,30], while the decreased expression has been found in ischemia-induced retina [29,30]. The intention of this study was to validate the results from previous studies and investigate the role of *miR-126* associated vascularization pathways in the laser-induced CNV mouse model, a well-established model which closely mimics the pathogenesis of AMD in human beings. The potential of *miR-126* to be used as a therapy was also investigated by restoring *miR-126 in vitro* and *in vivo*.

## 2. Results

### 2.1. Expression of miR-126 in Laser-Induced Choroidal Neovascularization (CNV) Mouse Model

**Figure 1:**
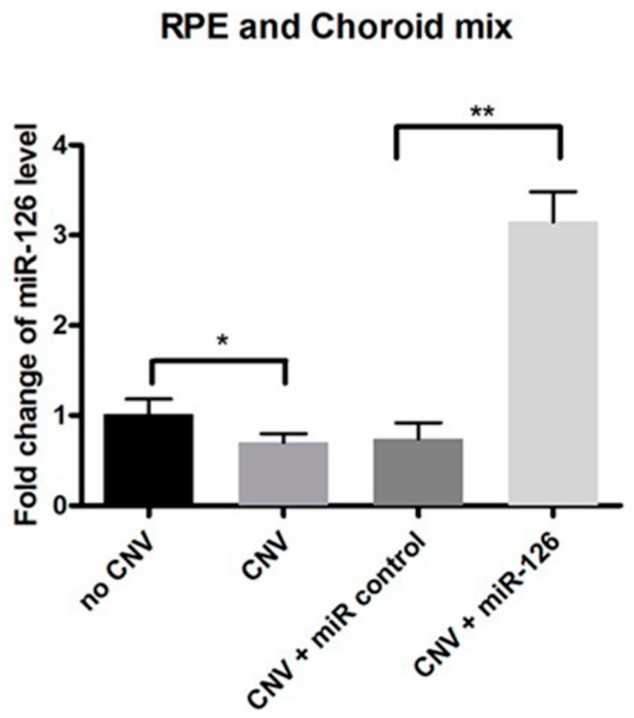
*miR-126* expression in choroidal neovascularization (CNV) mice. Fourteen days after transfection, the expression of *miR-126* in CNV mice eyes in the following treatment groups: (1) No laser-induced CNV (no CNV): Control mice without treatment; (2) CNV: Mice exposed to CNV laser treatment only, without transfection; (3) CNV + *miR-control*: Mice exposed to CNV laser treatment and transfected with negative control through an intravitreal injection; and (4) CNV + *miR-126*: Mice exposed to CNV laser treatment and transfected with miR-126 mimic through an intravitreal injection. Data are presented as relative fold change of *miR-126* level ± standard deviation (SD), *n* = 12, * *p* < 0.05, ** *p* < 0.01.

Fourteen days post transfection, the expression of *miR-126* in mice eyes was measured by qPCR (RPE/choroid mix *n* = 12). *miR-126* was significantly downregulated (*p* < 0.05) in the CNV eyes compared with the untreated eyes (Figure 1). CNV mice were transfected using a transfection agent coupled with either a *miR-126* mimic or a negative control oligonucleotide (*miR-control*). Successful *miR-126* transfection was confirmed using PCR analysis (*n* = 12, *p* < 0.01) after injection with miR-126 mimic (Figure 1). We demonstrated that the miR-126 remains effective even after 14 days of *in vivo* transfection.

### 2.2. Changes of miR-126 Target Genes in the Eyes of CNV Mice

**Figure 2:**
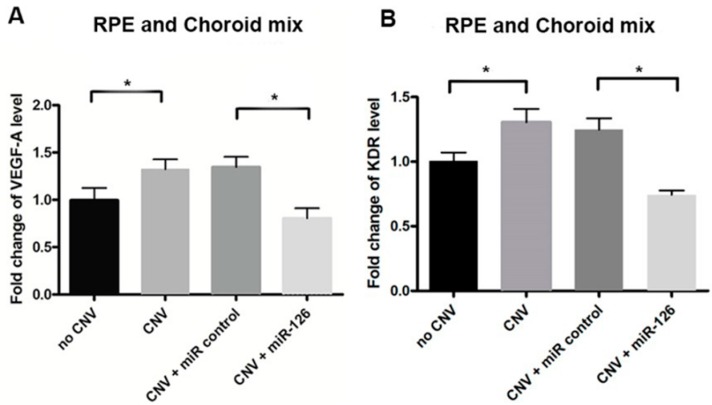

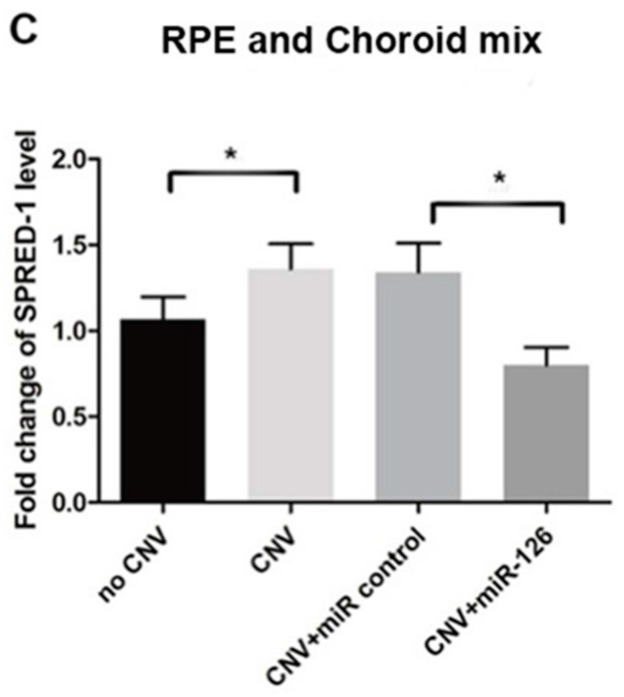
mRNA expression of *miR-126* target genes in the eyes of CNV mice. *VEGF-A*, *KDR* and *SPRED-1* mRNA expression in the eye tissue of control mice either transfected with a *miR-control* or a *miR-126* *mimic* through an intravitreal injection: (**A**) relative *VEGF-A* mRNA expression; (**B**) relative *KDR* mRNA expression; and (**C**) relative *SPRED-1* mRNA expression. Data are presented as mean fold change of mRNA levels ± SD, *n* = 12, * *p* < 0.01.

**Figure 3:**
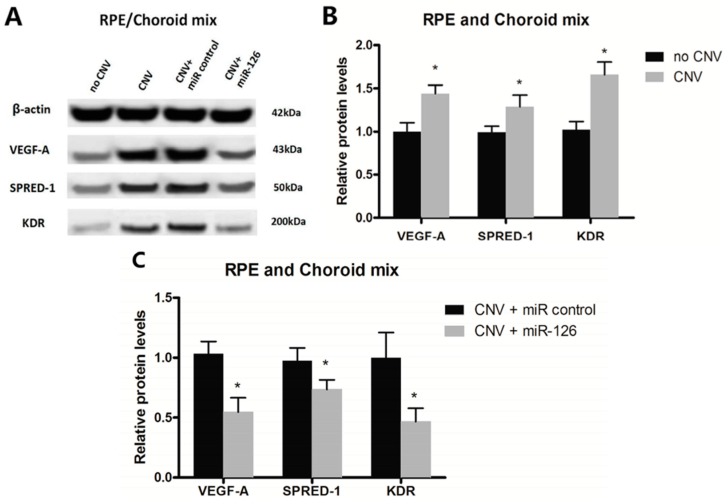
Changes of *miR-126* target genes protein level in the eyes of CNV mice: (**A**) Western blot expression pattern of *VEGF-A*, *KDR* and *SPRED-1* in different groups of mice with or without CNV, and CNV mice transfected with *miR-control* or *miR-126*; (**B**) quantification of relative target genes protein expression in no CNV and CNV groups; and (**C**) relative expression of target genes protein in a different group of CNV mice after transfection. Data are expressed as relative fold change of protein level ± SD, *n* = 12, * *p* < 0.05.

The downstream effect of reduced expression of miR-126 was assessed by qPCR and Western blot analysis. As shown, *VEGF-A*, *KDR* and *SPRED-1* mRNA expression were upregulated in the CNV mice eyes compared with untreated controls (*n* = 12, *p* < 0.05, *p* < 0.01) (Figure 2). Similarly, an increase in these targets protein levels was observed in the CNV mice compared with untreated ones. To further explore the effects of miR-126 *in vivo*, the expression of miR-126 was altered in the CNV mice to determine if restoration of miRNA expression would result in modulation in the expression of predicted mRNA and protein targets. CNV mice with overexpressed miR-126 exhibited a significant decrease in mRNA and protein expression of *VEGF-A*, *KDR* and *SPRED-1* (*n* = 12, *p* < 0.05), measured by qPCR and Western blot, respectively (Figure 2 and Figure 3). A clear functional impact of miR-126 regulated targets on CNV severity *in vivo* has been demonstrated.

### 2.3. Restoration of miR-126 Levels Reversed High mRNA and Protein Expression of Target Genes in Human Microvascular Endothelial Cell (HMEC)

**Figure 4:**
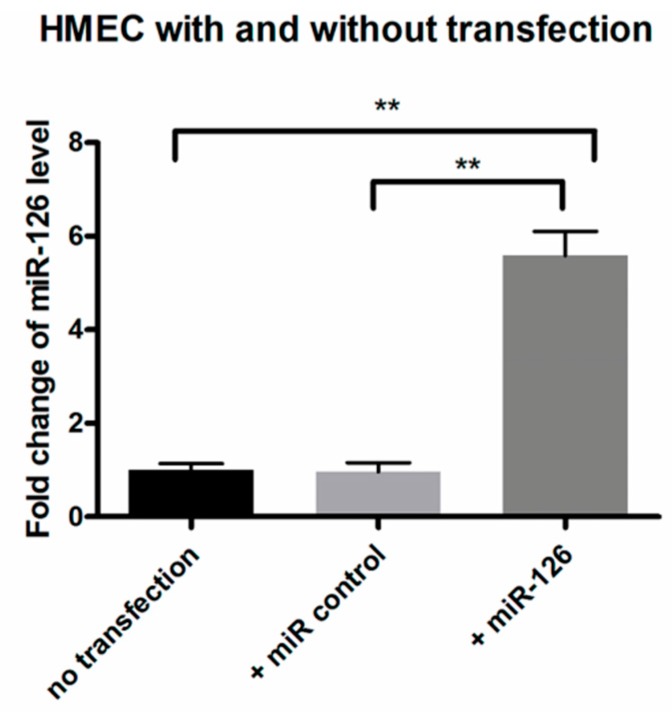
Expression of *miR-126* in human microvascular endothelial cell (HMEC) after transfection. *miR-126* expression in HMEC without transfection or transfected with either a negative control or *miR-126*. Data are expressed as relative fold change of *miR-126* ± SD (*n* = 4), ** *p* < 0.01.

**Figure 5:**
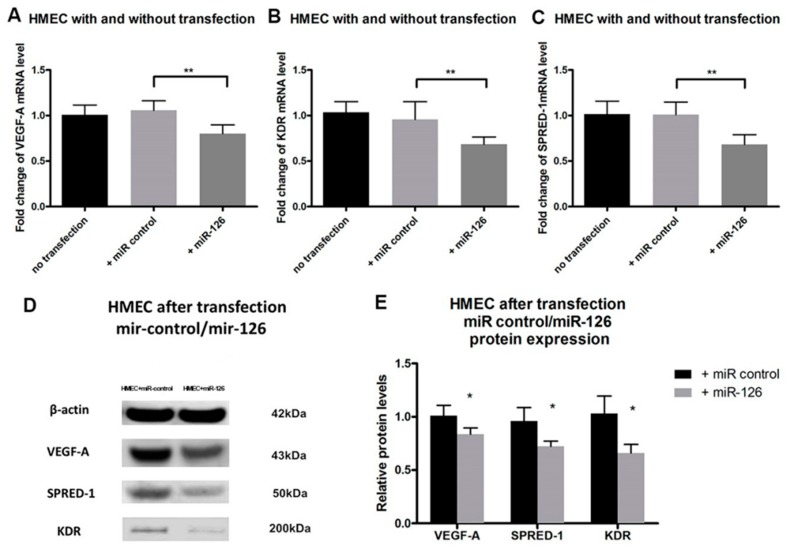
*miR-126* target genes expression in HMEC after transfection: (**A**) *VEGF-A*; (**B**) *KDR*; and (**C**) *SPRED-1* mRNA expression in HMECs was determined by qPCR in the following treatment groups: No transfection: HMECs without transfection; +*miR-control*: HMECs transfected with *miR-control*; +*miR-126*: HMECs transfected with *miR-126 mimic*. Data are presented as mean fold change of mRNA levels ± SD (*n* = 4), ** *p* < 0.01; (**D**) Western blot images of *miR-126* target genes protein in HMEC after transfection of either the negative control *miR-126*; and (**E**) Quantification of relative target genes protein expression in HMECs receiving *miR-control* or *miR-126* transfection determined by Western blot. Data are presented as mean fold change of protein levels ± SD (*n* = 4), * *p* < 0.05.

Overexpression of *miR-126* in HMECs inhibited both mRNA and protein expression of *VEGF-A*, *KDR* and *SPRED-1*. Transfection of *miR-126* in HMECs (Figure 4) resulted in decreased expression of *VEGF-A* and *KDR* mRNA (*n* = 4, *p* < 0.01) (Figure 5A,B), and *SPRED-1* mRNA expression was also downregulated (*n* = 4, *p* < 0.01) (Figure 5C). The expression of the respective proteins was also significantly reduced (*n* = 4, *p* < 0.05) (Figure 5D,E).

### 2.4. Transfection of miR-126 Decreased Tube Formation in HMECs

**Figure 6:**
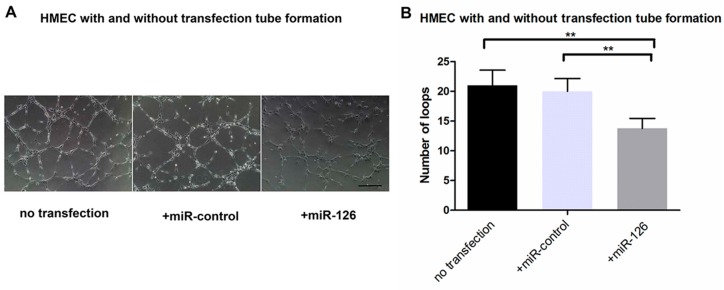
HMECs tube formation after transfection: (**A**) representative images of HMEC tube formation, without transfection and after transfected with *miR-control* or *miR-126 mimic*; Scale bar = 20 μm and (**B**) analysis of capillary-like tube formation of HMECs in different groups with no transfection, transfected *miR-control* or *miR-126 mimic*. Data are present as mean number of loops formed ± SD (*n* = 4), ** *p* < 0.01.

We performed tube formation after HMECs received scramble control miR or *miR-126 mimic* treatment. The number of loops in the tube formation assay showed a significant decrease (*n* = 4, *p* < 0.01) in the *miR-126* transfected HMECs compared to both the nontransfected and *miR-control* groups (Figure 6A,B). It highlights that miR-126 can inhibit the formation of capillary tubes.

### 2.5. Effect of miR-126 Overexpression on VEGF-Induced Migration of HMEC

**Figure 7:**
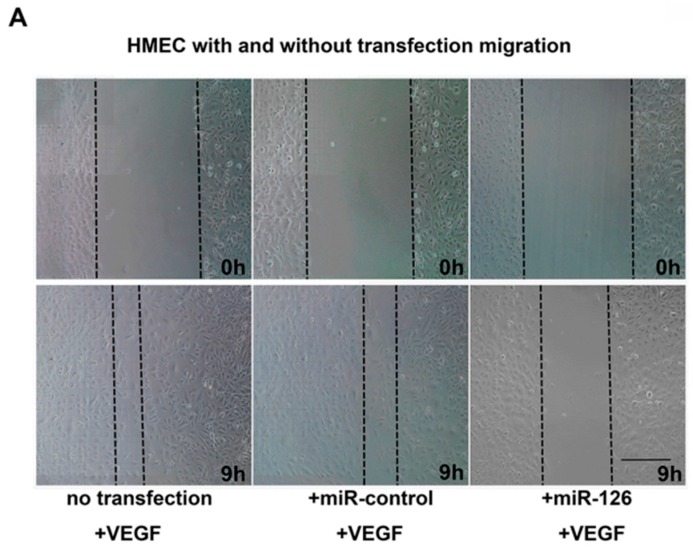

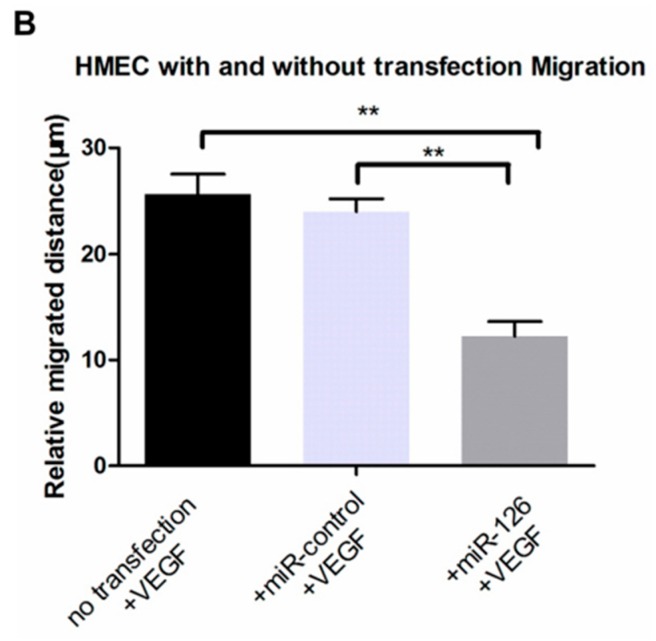
Inhibition of VEGF-induced cell migration by *miR-126* overexpression. (**A**) Representative images of HMEC migration in different groups at *t* = 0 h and *t* = 9 h; Scale bar = 20 μm and (**B**) analysis of migrated distance of HMECs with no transfection, transfected *miR-control* or *miR-126 mimic* group. Data are present as mean migrated distance ± SD (*n* = 4), ** *p* < 0.01.

Migration of endothelial cells is an essential step in the process of blood vessel growth. In order to better clarify the role of *miR-126* for HMEC migration, we used VEGF (50 ng/mL) as a stimulus. The distance of cells migration in scratch wound assay were significant decrease (*n* = 4, *p* < 0.01) in the *miR-126* transfected HMECs compared to both the nontransfected and *miR-control* groups (Figure 7A,B).

### 2.6. Restoration of miR-126 in Laser-Induced CNV Lesions in Mice Eyes

**Figure 8:**
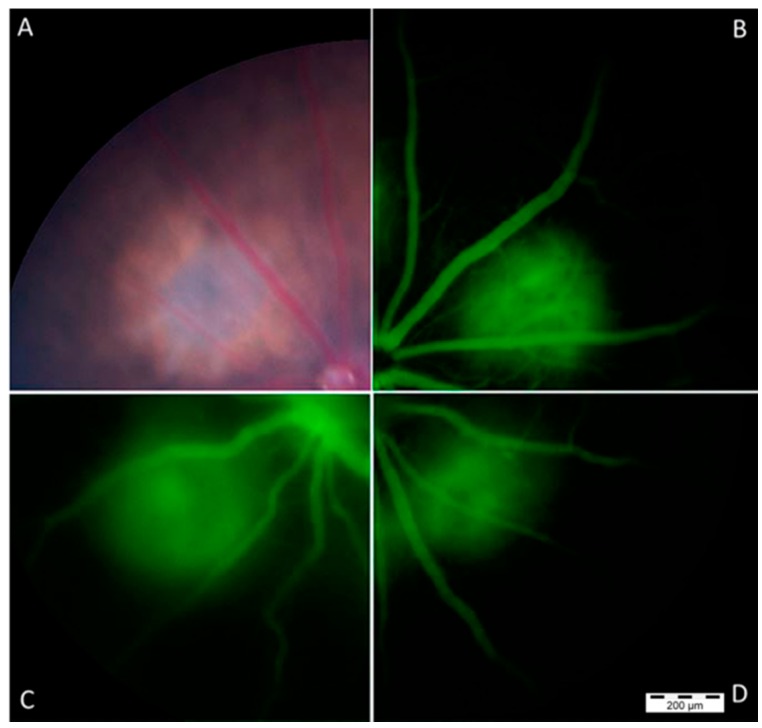
Representative fundus images of the mouse retina were taken after laser treatment: (**A**) a representative color fundus image of the mouse after laser-induced CNV treatment; (**B**) fluorescein angiography (FFA) of CNV lesion of mouse receiving CNV generation only; (**C**) FFA of mouse receiving CNV-generating laser treatment and *miR-control*; and (**D**) FFA of mouse receiving CNV-generating laser treatment and *miR-126 mimic*. Scale bar = 200 μm.

**Figure 9:**
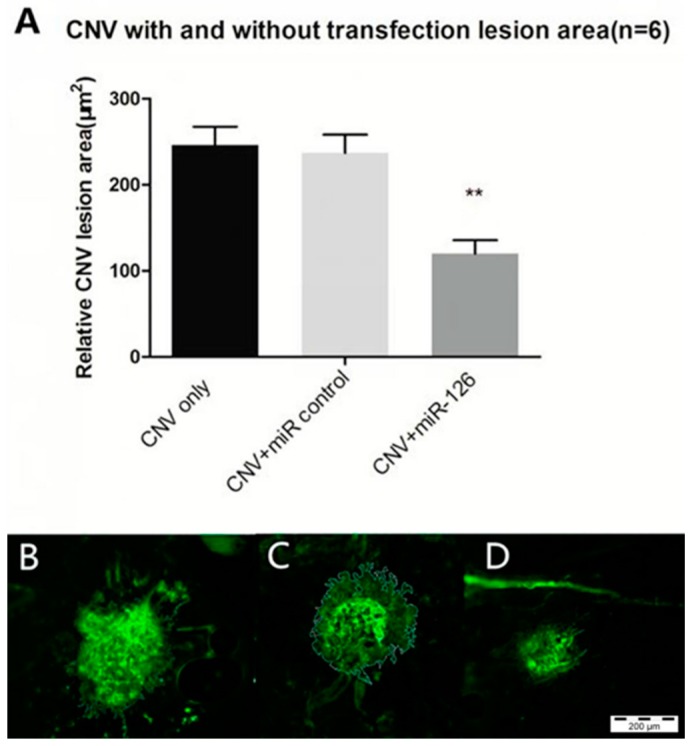
Representative micrographs of the CNV lesions from choroidal flatmount in three groups: (**A**) Quantification of relative 2D lesion areas in the eyes of CNV mice after laser treatment in choroidal flatmount. Lesion areas were measured using the ImageJ software. Data are expressed as relative CNV lesion area ± SD, *n* = 6, ** *p* < 0.01; (**B**–**D**) Representative micrographs of CNV lesions of Isolectin GS-IB4 *AF488 stained choroidal flatmount, with manual border delineation in the ImageJ: (**B**) CNV-only (CNV mice without treatment); (**C**) CNV+ *miR-control* (CNV mice transfected with negative control); and (**D**) CNV+ *miR-126* (CNV mice transfected with miR-126 *mimic*); Scale bar = 200 μm.

CNV lesion size and severity were determined to confirm the effect of the overexpression of *miR-126* on the CNV mice. Figure 8 shows that representative composite fundus images taken 1–2 weeks post treatment. In Figure 9, CNV mice transfection with the negative control miR showed no changes in CNV lesion area, but the mice treated with *miR-126 mimic* had significantly smaller CNV lesion areas. The findings of this study demonstrated that *miR-126* successfully reduced angiogenesis in the mice eyes.

## 3. Discussion

The endothelial-specific microRNA *miR-126* has been shown to play a role in regulating vascular integrity and angiogenesis as well as multiple disease processes [22,23]. However, the role of *miR-126* in AMD still remains unknown at present.

In this study, we found that *miR-126* was downregulated in the laser-induced CNV mice eyes. The decreased *miR-126* expression is associated with the increased mRNA and protein levels of *VEGF-A*, *KDR* and *SPRED-1*. Restoration of *miR-126* can reverse these increases in the CNV mouse model and inhibit CNV formation. HMECs transfected with *miR-126* *mimic* also showed an inhibition in mRNA and proteins levels of *VEGF-A*, *KDR* and *SPRED-1*. Upregulation of *miR-126* reduced endothelial cell tube formation and *VEGF*-induced migration. The results based on this study confirm the established knowledge of *miR-126* involvement in angiogenesis and reveal a critical role for *miR-126* in CNV.

Reported *miR-126* levels in vascular pathogenesis were variable in the literature. Studies conducted by Fish *et al.* and Wang *et al.* showed that *miR-126* normally promotes vessel formation and stability by “repressing the repressors” of *VEGF* signaling and that loss of *miR-126* inhibited vascular growth in the embryonic stem cells and embryos of mice and zebrafish [19,31]. From those studies, it would appear that *miR-126* enhanced angiogenesis. However, more recently, it has been found that *miR-126* levels were decreased in ocular neovascularization diseases such as diabetic retinopathy or retinopathy of prematurity [32,33]. In our study, we found that *miR-126* expression was substantially decreased in laser-induced CNV model—the well-established model mimicking the pathogenesis of neovascular AMD (Figure 1).

It is becoming evident that miRNAs expression and function are highly tissue-specific [34]. miRNAs regulate gene expression levels by several pre- and post-transcriptional mechanisms yielding diverse downstream effects in different cells based on their cellular environments and stages of life [15]. This may explain why there is such variability observed between studies utilizing different tissue samples, and disease models.

The different expression pattern of *miR-126* in ocular neovascularization may be partly due to the anatomical characteristics of the eye. The physical barriers such as the blood–retinal barrier separate the ocular tissues from systemic circulation [35] and thus create a unique ocular microenvironment, which is important to protect the visual integrity. Ocular immune privilege allows for systemic immunological tolerance to antigens encountered into the eye and restricts the formation of potentially damaging immune responses. This may also restrict the influence of local factors and downstream effects of miRNA and explain the different expression of *miR-126* in ocular diseases compared to other systemic disorders.

*VEGF-A* and its receptor *KDR*, are extensively involved in the stimulation of the *MAPK/ERK* cascade which is essential for cell proliferation and the growth of new blood vessels. In wet AMD patients, *VEGF-A* level is increased [36]. *VEGF* causes leaky, disorganized and uncontrolled growth of new blood vessels, which are prone to hemorrhaging and leakage of plasma into the subretinal space [37,38]. *SPRED-1* has been known to inhibit the phosphorylation and the activation of Raf, one of the intermediate signaling components in the *VEGF*-dependent *MAPK/ERK* pathway involved in angiogenesis, thus negatively regulate *VEGF* signaling [19,39,40]. Our experiment shows that the low expression of *miR-126* in CNV mice correlates with the increased mRNA and protein expression of *VEGF-A*, *KDR* and *SPRED-1* and that restoration of *miR-126* can inhibit these increases (Figure 2 and Figure 3).

Kamal *et al.* suggested that HMECs are an appropriate model for studies involving microvascular endothelial cells such as angiogenesis [41]. Costa *et al.* used HMECs to compare the multiple effects of bevacizumab and ranibizumab in ocular angiogenesis, they demonstrated that HMEC is probably a more accurate model to study the effects on the angiogenic process, given their capillary background, characteristic of angiogenic vessels [42]. HMECs were used here as the *in vitro* replication of the microvasculature resident in the eye. In cultured HMECs, we found that the decreased expression of *VEGF-A*, *KDR* and *SPRED-1* after *miR-126* transfection (Figure 5) were consistent with *in vivo* results. The changes in the target mRNA and protein levels suggest that *miR-126* not only represses translation of these mRNAs, but also causes the degradation of mRNA [8,29].

Our results were also consistent with findings from other studies, suggesting that *miR-126* can influence *VEGF* signaling to a certain extent, through *SPRED-1* and *PIK3R2* [39,43,44], or *VEGF-A* [31,32]. Although it has been suggested that *miR-126* targets *PIK3R2* as well [45], the concentration of *PIK3R2* mRNA and proteins in our samples were below the detection limit. The decreased expression of *KDR* after *miR-126* transfection suggested that *KDR* may be an indirect target of *miR-126* in the eye; alternatively, the *miR-126* mediated change in *VEGF-A* level may cause downstream changes in *KDR* expression (Figure 2 and Figure 3).

CNV development involves proliferation, differentiation, capillary formation and migration of endothelial cells. The significantly reduced tube formation and *VEGF-*induced migration were observed in *miR-126* transfected HMECs, further reinforces the notion that the increase in *miR-126* expression could inhibit and possibly reverse pathological angiogenesis in AMD (Figure 6 and Figure 7).

Based on the above findings, we postulate that restoring the *miR-126* levels could inhibit the progression of CNV in the eye and then confirmed this hypothesis in the following experiments. *miR-126 mimic* was administered via a transfection reagent by intravitreal injection into the laser induced CNV eyes. After successful transfection, the mice treated with *miR-126 mimic* had significantly smaller CNV lesion areas compared to the untreated “CNV only” group (*p* < 0.01), while *miR-control* group showed no significant difference in CNV lesion size compared to the controls (Figure 9).

The primary pathogenesis in wet AMD occurs in the outer retina, including the RPE, Bruch’s membrane, and choroid [46,47,48,49,50]. In our study, the alteration of miR-126 expression by miR-126 mimc injection in the eye may target also RPE. RPE cells are important in secreting angioregulatory proteins, including VEGF [51,52] and PEDF [53,54,55]. The balance between VEGF-A and PEDF plays an important role in choroidal neovascularization [54,56,57,58]. Masayuki *et al.* cultured RPE under hypothermia that exhibit decreased VEGF-A and sustained PEDF expression [59]. In our study, decreased VEGF-A may induced the increased PEDF that is secreted from RPE. Even if it is not a large amount, may act as an antagonist for VEGF-A, it may play a role in preventing AMD development conjunction with miR-126 during this situation. Moreover, a study indicated that primary RPE cells were shown to secrete and respond to placental growth factor (PlGF), a member of VEGF family. There is evidence that the retinal expression of PlGF mRNA is increased during diabetic retinopathy [60]. PlGF facilitates endothelial cell proliferation and vascular permeability by potentiating the activity of VEGF [61,62,63]. Our study showed that miR-126 overexpression can inhibit CNV to some extent by regulating VEGF-A, and PlGF might be a potential target of miR-126 in AMD.

In this study, we found that expression of *miR-126* was decreased in laser induced CNV mice; restoration of *miR-126 mimic* decreased *VEGF-A* levels and consequently influenced its downstream pathway and reduced CNV severity. *miR-126* seems to be a beneficial alternative to conventional *anti-VEGF* antibody for the treatment of wet AMD. However the CNV was not fully inhibited by *miR-126* transfection, indicating that there are other pathways involved in ocular neovascularization. Systemic delivery of engineered miRNA antagonists (antagomiRs) has been shown to effectively modulate angiogenesis with a long lived duration [64]. *miR-122* as a therapeutic agent has demonstrated good efficacy and outstanding safety profile in participants in the first Phase I trial and progressed to Phase II trials [65]. The potential of *miR-126* as a therapeutic target is promising and should be further explored for the treatment of AMD.

## 4. Experimental Section

### 4.1. Animals

All experiments were performed in accordance with the ARVO Statement for the Use of Animals in Ophthalmic and Vision Research, and approved by Harbin Medical University and the Royal Victorian Eye and Ear Hospital Animal Research & Ethics Committee (11-233, 5 March 2014). All mice were euthanized by CO_2_ inhalation and all efforts were made to minimize suffering.

#### 4.1.1. Establishment of Laser-Induced CNV Mouse Model

Adult C57BL/6 mice (9 weeks old) were maintained in a 12-h light/dark cycle with a room illuminance of 140–260 lux during the bright portion of the cycle. Animals were provided standard food and water *ad libitum*. Laser treatment was performed as described in a previous study [17]. CNV lesions were evaluated after laser treatment using Micron III, and choroidal flatmount. C57BL/6 mice were anesthetized through an intraperitoneal injection with a mixture of ketamine and xylazine (100 and 10 mg/kg, respectively) in a dosage of 0.1 mL/10 g of body weight. The pupils were dilated with a drop of 1% tropicamide solution (Alcon, Fort Worth, TX, USA), followed by 0.5% proxymetacaine hydrochloride (Alcon) as a topical anesthetic. Laser (50-µm spot size, 0.05 s duration, and 500 mW) was applied using a laser photocoagulator (532-nm wavelength) to the retina radially about the optic nerve with a coverslip as a contact lens. The formation of a small bubble at the laser spot indicated a successful rupture of the Bruch’s membrane and formation of CNV. Mice without the bubble formation or with vitreal hemorrhage obscuring the posterior segment were excluded. CNV lesions were evaluated after laser treatment using Micron III and histological staining of choroidal flatmount.

#### 4.1.2. Micron III Imaging

Anesthetized mice pupils were dilated with a drop of 1% tropicamide solution (Alcon). The mouse eyes were covered with a drop of ocular lubricating gel (Alcon), which served as an optical coupling medium, and the vascular network of the live CNV mice was imaged *in vivo* using the Micron III retinal imaging camera system (Phoenix Research Laboratories, Pleasanton, CA, USA). Fundus images of both eyes were obtained before a dose of 1 mL/10 g body weight of 1% fluorescein sodium was injected intraperitoneally into each mouse to obtain a fluorescence image [66].

#### 4.1.3. Choroidal Flatmount

To analyze the area of the CNV lesion, mice eyes were enucleated and fixed in 4% paraformaldehyde for 60 min. Under a dissecting microscope, the anterior segment and neuroretina were removed. The sclera–choroid–retinal pigment epithelial complex was isolated and incubated in a blocking solution (0.5% bovine serum albumin, 0.5% triton-X) for 4 h at 4 °C and then for 16 h at 4 °C in Isolectin GS-IB4 conjugated with Alexa Fluor 488 (1:100 dilution, 500 μg/mL). Fluorescent micrographs of the choroidal flatmount were then taken using an Olympus DP-71 camera system attached to an Olympus BX61 microscope (Olympus, Tokyo, Japan). The area (µm^2^) was measured using the ImageJ software (2x 2.1.4.7, National Institute of Mental Health, Bethesda, MD, USA). The measurements obtained from multiple lesions from the eyes of each animal were averaged.

#### 4.1.4. Intravitreal Injection of miRNA

Scrambled mirVana™ miRNA Mimic, Negative Control #1(Ambion, Foster City, CA, USA) or *miR-126* *mimic* (5′-UCGUACCGUGAGUAAUAAUGCG-3′, Ambion) at a concentration of 5 µM was complexed with Invivofectamine 2.0 Transfection Reagent (Invitrogen, Carlsbad, CA, USA) in accordance with the manufacturer’s instructions. The CNV mice were randomly divided into three groups (*n* = 12/group, and 24 eyeballs/group). Each group received either no treatment or an intravitreal injection of 5 µL of either the scramble control miR or *miR-126 mimic* treatment on Day 0 (immediately after laser treatment) and readministered on Day 7. For the intravitreal injection, a hole was first made by inserting a 30-gauge needle posterior to the limbus of the mice eye. Then the respective compounds were injected through the initial hole using a 34-gauge Hamilton syringe. Insertion and infusion were performed and directly viewed through an operating microscope. Care was taken not to injure the lens or the retina [31,67]. Mice were sacrificed on day 14 for choroidal flatmount, real-time qPCR and Western blot analysis.

### 4.2. Cells

Human microvascular endothelial cells (HMECs) were obtained from Hitesh Peshavariya of Centre for Eye Research Australia. HMECs were grown in Endothelial Basal Medium (EBM, Lonza, Basel, Switzerland), supplemented with EGM-MV SingleQuot Kit Supplement & Growth Factors (Lonza) at 37 °C in a humidified chamber at 5% CO_2_.

#### 4.2.1. *In Vitro* Transfection of HMECs

A 10 nM mimicking *miR-126* (Pre-miR miRNA Precursor Molecule, Ambion) or nonspecific control miRNA (Pre-miR Negative Control #1, Ambion) was transfected into the HMEC cell lines using Lipofectamine RNAiMAX (Invitrogen) according to the manufacturer’s instructions.

#### 4.2.2. Tube Formation Assay and Scratch Wound Migration Assay

The ability of HMECs to form capillary-like tubes in culture was assessed by adding 5 × 10^4^ cells to 24-well plate, which contained 200 μL of pre-gelled Matrigel (BD Biosciences, San Jose, CA, USA) in 1 mL of a complete medium. Tube formation in the HMEC culture was quantified at *t* = 9 h by counting all tube-like structures in the gel under an inverted phase contrast microscope (Olympus IX71, Tokyo, Japan) at × 40 magnification. The data was presented as the total number of loops formed [68]. The migration of endothelial cells was monitored by generating a “scratch” assay. After 48 h of transfection, 3 × 10^4^ HMECs were plated in a 48-well plate previously labeled with lines to facilitate identification of the same regions at different time points. Cells were incubated for 48 h to reach confluence, and a scratch was made in the monolayer using a 200 µL pipette tip. Floating cells were removed by two gentle washes with phosphate buffered saline (PBS) and the growth medium was replaced with Endothelial Basal Medium only, with the addition of 50 ng/mL VEGF. Reference photographs were taken using a phase contrast microscope (Olympus IX71) and the photographed in the same areas at *t* = 0 h and 9 h. Endothelial cell migration was quantified as the difference in size between the denuded area immediately after the scratch and at *t* = 9 h.

### 4.3. RNA Preparation and qPCR

In mice, total mRNA was extracted using TRIzol (Invitrogen) from RPE/Choroids of both the control group and CNV group 14 days after the CNV laser procedure. In cells, the total mRNA and miRNA were isolated 48 h after transfection using the same method. The *miR-126* first-strand cDNA was prepared from 1 μg of RNA from each sample via RT-PCR with random primers or with *miR-126* and *U6* primers using a TaqMan MicroRNA reverse transcription kit (Applied Biosystems, Foster City, CA, USA). Quantitative qPCR was performed using the TaqMan MicroRNA assay (Applied Biosystems), with *U6* as the housekeeping gene. The qPCR analysis of mRNA was performed using a High-Capacity cDNA Reverse Transcription Kit (Applied Biosystems) and TaqMan gene expression master mix (Applied Biosystems) for *VEGF-A*, *KDR* and *SPRED-1*, with *18s* as the housekeeping gene. Each PCR reaction was performed in triplicates in a 10 µL volume using 5 µL master mix (Applied Biosystems) for 2 min at 50 °C followed by 45 cycles of 95 °C for 10 min, 95 °C for 15 s and 60 °C for 60 s in a real-time PCR system (Applied Biosystems). The results were expressed as Δ*C*_t_, and fold changes in relative mRNA expression were calculated using the equation 2^−ΔΔ*C*t^ [69]. All assays were performed according to the manufacturer’s instructions.

The sequences of the primes used are list as following: *miR-126:* mature sequence, 5′-UCGUACCGUGAGUAAUAAUGCG-3′. *U6*: mature sequence, 5′-GTGCTCGCTTCGGCAGCACATATACTAAA-3′. *VEGF-A:* forward 5′-GATCATGCGGATCAAACCTC-3′; reverse 5′-CCTCCGGACCCAAAGTGCTC-3′ *KDR:* forward 5′-GCGAAAGAGCCGGCCTGTGA-3′; reverse 5′-TCCCTGCTTTTGCTGGGCACC-3′; *SPRED-1:* forward 5′-GGCCTAGACATTCAGAGCCG-3′; reverse 5′-TAGTCTCATCCCCACAGTGG-3′; *18S*: forward 5′-GAAGGTGAAGGTCGGAGTC-3′; reverse, 5′-GAAGATGGTGATGGGATTTC-3′.

### 4.4. Western Blotting Analysis

The ocular tissues (RPE and choroid mix) were isolated from enucleated eyes under dissecting microscope and homogenized in RIPA buffer containing a protease inhibitor cocktail (1:100 dilution, Sigma-Aldrich, St. Louis, MO, USA). Cells were collected and lysed as above using RIPA buffer. The lysates were cleared by centrifugation at 12,000× *g* for 20 min at 4 °C. The protein concentration of the lysate was determined using the bicinchoninic acid (BCA) assay. Equal amounts of proteins (30 µg *in vivo* and 20 µg *in vitro*) were separated by SDS-PAGE gels (Invitrogen) and transferred onto polyvinylidene fluoride (PVDF) membranes (GE Healthcare, Little Chalfont, UK). After blocking with 5% nonfat milk powder in Tris-buffered saline (pH 7.5), the membranes were incubated with *anti-VEGFA* (ab51745, Rabbit polyclonal to *VEGF-A*, Abcam Sapphire Bioscience, Cambridge, MA, USA), *anti-SPRED-1* (ab77079, Rabbit polyclonal to *SPRED-1*, Abcam Sapphire Bioscience), *anti-KDR* (ab39256, Rabbit polyclonal to VEGF Receptor 2, Abcam Sapphire Bioscience) and *anti-β-actin* (ab3280, Mouse monoclonal to *Actin*, Abcam Sapphire Bioscience). After incubation with the horseradish peroxidase–conjugated secondary *IgG* (1:10,000, Sigma, USA), blots were developed and quantified with Odyssey Infrared Imaging System (Li-Cor Biosciences, Lincoln, NE, USA).

### 4.5. Statistical Analysis

All experiments were repeated a minimum of three times, and the error bars on graphs represent the mean ± SD, unless otherwise stated. All data were analyzed using Prism 6.0 (GraphPad Software, San Diego, CA, USA). miR-126 and its target genes mRNA expression, CNV lesion area, HMECs tube formation and migration were determined by ANOVA, a *p*-value of <0.05 was considered as significant. The expression of miR-126 target genes protein was analyzed by two tailed Student’s *t*-test with *p* < 0.05 declared significant.
